# Complete Plastome Sequence of *Ludwigia octovalvis* (Onagraceae), a Globally Distributed Wetland Plant

**DOI:** 10.1128/genomeA.01274-16

**Published:** 2016-11-17

**Authors:** Shih-Hui Liu, Christine Edwards, Peter C. Hoch, Peter H. Raven, Janet C. Barber

**Affiliations:** a Department of Biology, Saint Louis University, St. Louis, Missouri, USA; b Missouri Botanical Garden, St. Louis, Missouri, USA

## Abstract

Here, we present the first plastome of *Ludwigia octovalvis* (Onagraceae, Myrtales) as well as the first plastome in the subfamily Ludwigioideae. This genome is notable for its contracted inverted repeat regions and an expanded small single-copy region compared to other species in the orders Myrtales and Geraniales.

## GENOME ANNOUNCEMENT

*Ludwigia octovalvis* is distributed worldwide (1) and has a long history of usage for medicinal purposes and as herbal teas (2, 3). Presently, *L. octovalvis* is being explored in several biotherapy and anticancer studies (4–6). In some wetland ecosystems, *L. octovalvis* is also considered an invasive plant (7, 8). The plastome sample reported here was acquired from *L. octovalvis* seedlings (voucher: Liu 2014GH96, MO) growing in the research greenhouse at the Missouri Botanical Garden (St. Louis, MO, USA) from seeds collected at Angra dos Reis, Brazil (voucher: Martins & Liu 652, MO).

The library of genomic DNA was prepared using the NEBNext Ultra library prep kit and sequenced with a 2 × 150-bp Illumina MiSeq run. Trimming and quality control were conducted using Cutadapt (9) and BBDuk in BBMap (https://sourceforge.net/projects/bbmap/), respectively. To identify reads originating from the plastome of *L. octovalvis*, we mapped the trimmed reads to the plastomes of 14 species in orders Myrtales and Geraniales (accession numbers KC180806, KC180805, KC180804, KC180801, KP015033, KC180773, KC180782, KC180771, JQ809470, NC029808, KX118607*,* KX118606, KC180807, and GQ870669) and then extracted the matching reads using BWA (10) and SAMtools (11). The matching reads were then assembled *de novo* using SPAdes (12). Gaps among contigs were closed using an iterative mapping strategy (13) in which the contigs were extended by repeatedly mapping all trimmed reads to the contigs using the medium sensitivity/fast option in Geneious R7 (14). Sequences at the gaps and at the junctions between large single-copy (LSC)/small single-copy (SSC) regions and inverted repeats (IRs) were validated by visually inspecting the mapped reads. Gene annotations were transferred from the plastomes of three closely related species (accession numbers NC029808, KX118607, and KX118606) with a 60% or greater similarity in Geneious and then verified by BLAST searches. The tRNA genes were further confirmed using tRNAscan-SE 1.21 (15, 16).

The completed plastome of *L. octovalvis* is a circular molecule of 159,396 bp in length (198-fold coverage) with a G+C content of 37.4%. In total, 132 genes were annotated: 85 unique genes (including 22 tRNA genes) are located in the LSC, 13 unique genes (including one tRNA gene) are in the SSC, and 17 genes (including 4 rRNA genes and 7 tRNA genes) are in both IRs. Introns were detected in 17 genes (including 6 tRNA genes). No genes spanned across the LSC/SSC and IRs junctions. The remarkable features of this plastome are its considerable IR contraction and SSC expansion in comparison to other plastomes in the orders Myrtales and Geraniales; namely, the IR of *L. octovalvis* is 24.755 kb, whereas the IR of other species in the Myrtales and Geraniales is 25.7 to ~28.7 kb, and the SSC of *L. octovalvis* is 19.703 kb, in contrast to other species in the Myrtales and Geraniales that have an SSC of 4.5 to ~19.0 kb (17, 18).

These plastome data will be useful for investigating genome rearrangements in basal lineages in the Rosids II clade as well as for phylogenomic studies of Onagraceae and *Ludwigia*.

### Accession number(s).

The annotated plastome sequence of *L. octovalvis* was deposited in GenBank under accession no. KX827312.
