# Correction for Liu et al., Complete Plastome Sequence of *Ludwigia* *octovalvis* (Onagraceae), a Globally Distributed Wetland Plant

**DOI:** 10.1128/genomeA.00188-17

**Published:** 2017-03-09

**Authors:** Shih-Hui Liu, Christine Edwards, Peter C. Hoch, Peter H. Raven, Ahmad Rajeh, Janet C. Barber

**Affiliations:** a Department of Biology, Saint Louis University, St. Louis, Missouri, USA; b Missouri Botanical Garden, St. Louis, Missouri, USA; c Department of Mathematics and Computer Science, Saint Louis University, St. Louis, Missouri, USA

## AUTHOR CORRECTION

Volume 4, no. 6, e01274-16, 2016, https://doi.org/10.1128/genomeA.01274-16. Page 1: The byline and affiliation line should read as given above.

Page 1: Lines 3 and 4 of the Acknowledgments should read “ … and E. Colletti for growing plants from seeds.”

